# The Complexity of Online Patient Education Materials About Interventional Neuroradiology Procedures Published by Major Academic Institutions

**DOI:** 10.7759/cureus.34233

**Published:** 2023-01-26

**Authors:** Assala Aslan, Basel Musmar, Ahmed Mamilly, Amro Saad Aldine, Luis De Alba, Octavio Arevalo, Chaitanya Ahuja, Hugo H Cuellar

**Affiliations:** 1 Radiology, Louisiana State University, Shreveport, USA; 2 Medicine, An-Najah National University, Nablus, PSE; 3 Radiology, University of Texas Southwestern Medical Center, Dallas, USA; 4 Neuroradiology, Louisiana State University Health Sciences Center, Shreveport, USA; 5 Neuroradiology, Louisiana State University Health Sciences Shreveport, Shreveport, USA; 6 Radiology, Louisiana State University Health Sciences Center, Shreveport, USA

**Keywords:** education, patient, readability, radiology, interventional

## Abstract

Introduction

Health literacy is an independent predictor of population health status and is directly related to the readability of available patient education material. The National Institutes of Health (NIH) and the American Medical Association have recommended that patient education materials (PEMs) be written between a fourth- and a sixth-grade education level. The authors assessed the readability of online PEMs about neurointerventional procedures that have been published by several academic institutions across the US.

Methods

Online PEMs regarding five common neurointerventional procedures, including mechanical thrombectomy for large vessel occlusion, cerebral diagnostic angiography, carotid artery stenting, endovascular aneurysm embolization, and epidural steroid injection collected from the websites of 20 top institutions in Neurology and Neurosurgery. The materials were assessed via five readability scales and then were statistically analyzed and compared to non-institutional education websites (Wikipedia.com and WebMD.com).

Results

None of the PEMs were written at or below the NIH's recommended 6th-grade reading level. The average educational level required to comprehend the texts across all institutions, as assessed by the readability scales, was 10-11^th^ grade level. Some materials required a college-level education or higher. Material from non-institutional websites had significantly lower readability scores compared to the 20 institutions.

Conclusions

Current PEMs related to neurointerventional procedures are not written at or below the NIH's recommended fourth- to sixth-grade education level. Given the complexity of those procedures, significant attention should be pointed toward an improvement in the available online materials.

## Introduction

Approximately 80 million Americans have limited health literacy, which puts them at greater risk for poorer access to care and poorer health outcomes [1]. Literacy, which reflects the ability to read and comprehend information, is directly related to readability, which is the ease with which a text can be read and understood [2]. Within the US general population, the average reading level has been found to be that of a seventh- or eighth-grade student, prompting the National Institutes of Health (NIH) and the American Medical Association (AMA) to recommend that patient education materials (PEMs) be written at a reading level no higher than the sixth grade [3]. In this study, we aim to evaluate the readability of online PEMs related to common neurointerventional procedures.

This article was previously presented as a meeting abstract at the 2022 SNIS 19th Annual Meeting in Toronto.

## Materials and methods

Five common neurointerventional procedures were selected, including mechanical thrombectomy for large vessel occlusion, cerebral diagnostic angiography, carotid artery stenting, endovascular aneurysm embolization, and epidural steroid injection. The top 20 universities in Neurosurgery and Neurology based on the 2021 US News ranking were identified, and their websites were searched for online patient education material related to those procedures. Similarly, Wikipedia.com and WebMD.com, being common sources of non-institutional online patient education, were searched for similar materials for comparison purposes (Table 1).

**Table 1:** Top 20 institutions for Neurology and Neurosurgery according to US News, which were included in this study.

	Institution
1	New York University - Langone Hospitals
2	University of California in San Francisco Medical Center
3	New York-Presbyterian Hospital-Columbia and Cornell
4	Rush University Medical Center
5	Johns Hoskins Hospital
6	Mayo Clinic-Rochester
7	Cedars-Sinai Medical Center
8	Cleveland Clinic
9	Mount Sinai Hospital
10	Northwestern Memorial Hospital
11	Massachusetts General Hospital
12	University of California in Los Angeles Medical center
13	Barnes-Jewish Hospital
14	Stanford Hospital
15	University of Pennsylvania
16	Houston Methodist Hospital
17	Long Island Jewish Medical Center - Northwell Health
18	Brigham and Women’s Hospital
19	University of Michigan Hospitals
20	University of Texas Southwestern Medical Center

The text was reformatted using word-processing software (Microsoft Word, Microsoft Corp.) to exclude any navigational cues, hyperlinks, references, and images. An online readability analysis through WebFX Readability (https://www.webfx.com/tools/read-able) was used to assess the readability of the educational materials, using a series of five established scales: Flesch Reading Ease, Gunning Fog, Flesch-Kincaid, Coleman-Liau, Simple Measure of Gobbledygook (SMOG), and Automated Readability Index (Table 2).

**Table 2:** Readability assessment scales The letters designate the following: A = average number of letters per 100 words; B = average number of sentences per 100 words; C = number of “easy” words within sample; D = average number of words per sentence; E = average number of sentences; F = number of polysyllabic words in sample; G = number of characters (letters and numbers).

Test	Formula
Coleman Liau Index	((0.0588 × A) − (0.296 × B)) − 15.8
Flesch Kincaid Reading Ease	206.835 – ((1.015 × C) – (84.6 × D))
Gunning Fog Score	0.4 × ((D ÷ E) + 100 (G ÷ D))
Flesch Kincaid Grade Level	(0.39 × C) + (11.8 × D) − 15.59
SMOG Index	1.043 × √(F × (30/E)) + 3.1291
Automated Readability Index	4.71 × (G ÷ D) + 0.5 (D ÷ E) – 21.43

SPSS version 26.0 (IBM Corp., Armonk, NY) was used to perform statistical analyses. The Kruskal-Wallis test was used to compare the average readability scores across the various scales and websites. Statistical significance was defined by a p-value < 0.05.

## Results

The 20 included universities had a total of 76 online PEMs related to the studied procedures, including thrombectomy (n = 9), angiography (n = 17), carotid stent (n = 16), aneurysm embolization (n = 15), and epidural steroid injection (n = 19). Two additional materials on each procedure were obtained from Wikipedia.com and WebMD.com.

**Figure 1:**
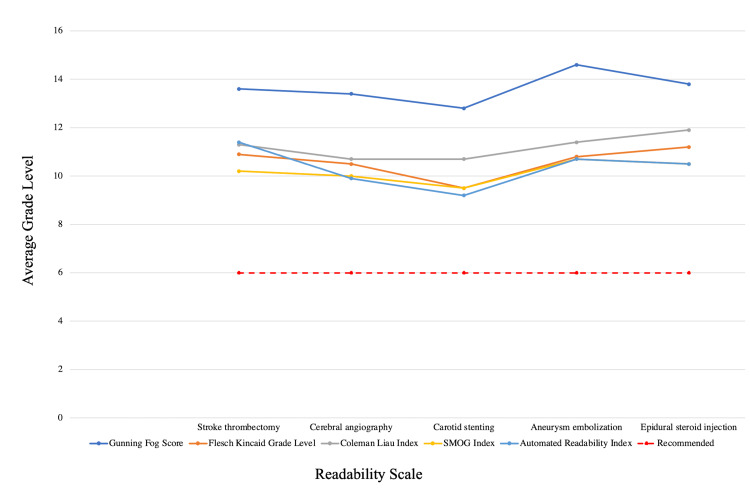
Graph showing the mean readability scores across different neurointerventional procedures compared to the recommended level by AHA/NIH.

For each procedure, the readability scores from different tests compared to the recommended level by AMA and NIH are illustrated in Figure 1.

The means and standard deviation for various readability scores of each procedure are reported in Table 3.

**Table 3:** Readability scores of online patient education materials for different procedures

Procedure	Gunning Fog Score	Flesch Kincaid Grade Level	Coleman Liau Index	SMOG Index	Automated Readability Index	Mean	Flesch Kincaid Reading Ease
Stroke thrombectomy	13.6 ± 5.2	10.9 ± 4.8	11.3 ± 3.4	10.2 ± 3	11.4 ± 6.1	11.5 ± 3.9	50.9 ± 17.2
Cerebral angiography	13.4 ± 3	10.5 ± 2.5	10.7 ± 2.2	10 ± 2.1	9.9 ±3	10.9 ± 2.3	50.7 ± 13.4
Carotid stenting	12.8 ± 2.4	9.5 ± 1.8	10.7 ± 2.2	9.5 ± 1.6	9.2 ± 2	10.3 ± 1.6	55.5 ± 10.9
Aneurysm embolization	14.6 ± 3.4	10.8 ± 2.7	11.4 ± 2	10.7 ± 2.2	10.7 ± 3.1	11.6 ± 2.3	49.7 ± 11.5
Epidural steroid injection	13.8 ± 4.2	11.2 ± 3.9	11.9 ± 2.8	10.5 ± 3	11.1 ± 4.5	11.7 ± 3.4	47 ± 18.2

Carotid stents had the lowest mean readability score (easiest to comprehend) while epidural steroid injection had the highest score (hardest to comprehend). There was no significant difference in the mean readability score between the universities (p = 0.07). Conversely, the mean readability scores were significantly lower on Wikipedia.com and WebMD.com as compared to the universities’ online material (9.4 vs 11.4, p = 0.016) (Table 4).

**Table 4:** Comparing readability scores of online patient education materials between universities and Wikipedia.com/WebMD.com

Source	Gunning Fog Score	Flesch Kincaid Grade Level	Coleman Liau Index	SMOG Index	Automated Readability Index	Mean		Flesch Kincaid Reading Ease
Universities	14.3 ± 3.2	10.9 ± 3.1	10.7 ± 2.1	10.6 ± 2.2	10.9 ± 3.7	11.4 ± 2.7	P = 0.016	51.2 ± 14.1
Wikipedia.com and WebMD.com	8.9 ± 3.1	8.3 ± 2.4	15.5 ± 1.8	7.2 ± 1.6	7 ± 2.7	9.4 ± 2.2		45.9 ± 16.6

## Discussion

The evolution of the internet has made it a major source of self-education for the public and allowed easier access to a wealth of online information. Currently, patients are increasingly using online PEMs for health information, with approximately 35% of American adults searching for diagnoses online. Readily accessible PEMs have been shown to increase healthcare utilization, preventative care, and patient-physician communication by providing a better understanding of diagnoses, symptomatology, treatment options, rehabilitation, and recovery, leading to improved health literacy [4-6].

Literacy has been touted as the single best predictor of healthcare status [5]. Poor health literacy is estimated to contribute to more than 73 billion dollars of additional burden to the US healthcare system [1,7]. The healthcare cost of Medicaid patients with limited literacy is about four times that of those who have adequate health literacy [8], as poor health-literate patients are assumed to be at high risk of exacerbation of their health problems, which in turn contributes to rising healthcare costs [9]. This burden, as well as the communication gap between patients and doctors, increases with the increased complexity of procedures, including neurointerventional procedures.

In this study, the mean readability scores for material published in major universities for the most common neurointerventional procedures were at 10-11th-grade level, which are 4-5 grades higher than the recommended level by AMA and NIH. Some of those materials were only comprehendible for the college level of education. When those materials were compared to similar materials obtained from non-institutional sources like Wikipedia.com and WebMD.com, the latter two had significantly lower readability scores, making them more comprehensible to the public. However, in a study by Dutta-Bergman et al. which evaluated the trustworthiness of different sources of healthcare information on the internet, nearly half of the respondents revealed that they considered medical universities as one of the most trusted sources of online PEMs [10]. Yet, there remains a considerable shortcoming in those available materials. Furthermore, even websites that provide high-quality, evidence-based information, need to be written at a level appropriate for patients without extensive medical knowledge. In addition to quality, the readability of PEMs is an important consideration to ensure patients can understand and apply information related to their conditions and treatments.

Besides improving the general health of the public and the understanding of pathology and treatment, improving the readability of PEMs may also indirectly serve an important role in decreasing the frequency with which malpractice litigation occurs [2]. Improving the readability of PEMs could be achieved by minimizing the use of complex words, decreasing the number of words per sentence and syllables per word, using numbering or bullet points, and writing in an active voice [11]. Subsequently, this will lead to a better understanding of the procedure context and outcomes by the patients which will then improve the relationship between them and their interventionalists.

Limitations

This study is limited by the small number of institutions and materials included. It also does not consider other factors that can contribute to online material comprehension, including socioeconomic status, race, education, and income which can all contribute.

## Conclusions

Online PEMs help improve healthcare utilization, preventative care, and patient-physician communication. However, current materials related to neurointerventional procedures are not written at or below the NIH's recommended fourth- to sixth-grade education level. This would impact the utility of those PEMs for the general public. Given the complexity of those procedures, significant attention should be pointed towards an improvement in the available online materials by minimizing the use of complex words, decreasing the number of words per sentence and syllables per word, using numbering or bullet points, and writing in an active voice.

## References

[1] Berkman ND, Sheridan SL, Donahue KE, Halpern DJ, Crotty K (2011). Low health literacy and health outcomes: an updated systematic review. Ann Intern Med.

[2] Gupta R, Adeeb N, Griessenauer CJ (2017). Evaluating the complexity of online patient education materials about brain aneurysms published by major academic institutions. J Neurosurg.

[3] Walsh TM, Volsko TA (2008). Readability assessment of internet-based consumer health information. Respir Care.

[4] Mazmudar RS, Sheth A, Tripathi R, Scott JF (2021). Readability of online Spanish patient education materials in dermatology. Arch Dermatol Res.

[5] Bass L (2005). Health literacy: implications for teaching the adult patient. J Infus Nurs.

[6] Punia V, Dagar A, Agarwal N, He W, Hillen M (2014). Comparison of neurological healthcare oriented educational resources for patients on the internet. J Clin Neurosci.

[7] Badarudeen S, Sabharwal S (2010). Assessing readability of patient education materials: current role in orthopaedics. Clin Orthop Relat Res.

[8] Weiss BD, Blanchard JS, McGee DL, Hart G, Warren B, Burgoon M, Smith KJ (1994). Illiteracy among Medicaid recipients and its relationship to health care costs. J Health Care Poor Underserved.

[9] Palumbo R (2017). Examining the impacts of health literacy on healthcare costs. An evidence synthesis. Health Serv Manage Res.

[10] Dutta-Bergman M (2003). Trusted online sources of health information: differences in demographics, health beliefs, and health-information orientation. J Med Internet Res.

[11] Grose EM, Holmes CP, Aravinthan KA, Wu V, Lee JM (2021). Readability and quality assessment of internet-based patient education materials related to nasal septoplasty. J Otolaryngol Head Neck Surg.

